# Self-Rated Health Among Italian Immigrants Living in Norway: A Cross-Sectional Study

**DOI:** 10.3389/fpubh.2022.837728

**Published:** 2022-06-01

**Authors:** Laura Terragni, Alessio Rossi, Monica Miscali, Giovanna Calogiuri

**Affiliations:** ^1^Department of Nursing and Health Promotion, Faculty of Health Sciences, Oslo Metropolitan University, Oslo, Norway; ^2^Department of Computer Science, University of Pisa, Pisa, Italy; ^3^Department of Historical and Classical Studies at the Norwegian University of Science and Technology, Trondheim, Norway; ^4^Department of Nursing and Health Sciences, Center for Health and Technology, Faculty of Health and Social Sciences, University of South-Eastern Norway, Drammen, Norway; ^5^Department of Public Health and Sport Sciences, Faculty of Health and Social Sciences, Inland Norway University of Applied Sciences, Elverum, Norway

**Keywords:** self-rated health, Italian immigrants, new mobilities, healthy immigrant effect, intra-European migration, health literacy, acculturation, machine learning

## Abstract

**Background:**

Most studies on immigrant health focus on immigrant groups coming from extra-European and/or low-income countries. Little attention is given to self-rated health (SRH) in the context EU/EEA migration. To know more about health among European immigrants can provide new insights related to social determinants of health in the migration context. Using the case of Italian immigrants in Norway, the aim of this study was to (i) examine the levels of SRH among Italian immigrants in Norway as compared with the Norwegian and the Italian population, (ii) examine the extent to which the Italian immigrant perceived that moving to Norway had a positive or negative impact on their SRH; and (iii) identify the most important factors predicting SRH among Italian immigrants in Norway.

**Methods:**

A cross-sectional survey was conducted among adult Italian immigrants in Norway (*n* = 321). To enhance the sample's representativeness, the original dataset was oversampled to match the proportion of key sociodemographic characteristics of the reference population using the ADASYN method (oversampled *n* = 531). A one-sample Chi-squared was performed to compare the Italian immigrants' SRH with figures on the Norwegian and Italian populations according to Eurostat statistics. A machine-learning approach was used to identify the most important predictors of SRH among Italian immigrants.

**Results:**

Most of the respondents (69%) rated their SRH as “good” or “very good”. This figure was not significantly different with the Norwegian population, nor to the Italians living in Italy. A slight majority (55%) perceived that their health would have been the same if they continued living in Italy, while 23% perceived a negative impact. The machine-learning model selected 17 variables as relevant in predicting SRH. Among these, Age, Food habits, and Years of permanence in Norway were the variables with the highest level of importance, followed by Trust in people, Educational level, and Health literacy.

**Conclusions:**

Italian immigrants in Norway can be considered as part of a “new mobility” of high educated people. SHR is shaped by several interconnected factors. Although this study relates specifically to Italian immigrants, the findings may be extended to other immigrant populations in similar contexts.

## Introduction

### Conceptualizing and Measuring Health

Health is a broad concept that embodies a wide range of meanings. The World Health Organization (WHO) defines health as “a state of complete physical, mental and social well-being and not merely the absence of disease or infirmity” (1). Several “objective” indicators of health (e.g., life expectancy, mortality rate, and incidence of diseases such as diabetes and heart diseases) are used in epidemiological studies (2). However, in the past two decades, Self-rated health (SRH) has increasingly gained popularity as a health indicator (3–6). SRH expresses an overall subjective evaluation by the respondents of their health and is often measured through simple (single-items) survey instruments (7). This instrument provides an overview of the general health status, health inequalities, and health care needs of the population. SRH has been adopted in surveys conducted at the European and national levels (3, 8). In Norway, SRH is used in the “survey on living conditions” conducted annually among the general Norwegian population (9) and was also conducted among the immigrant population in 2016 (10). Although SRH is a subjective measure, several studies indicate an association between SRH and clinical conditions. Single-item assessments of SRH, in its different variations, have shown high reliability in population studies (11). Moreover, it was found to be a valid predictor of mortality (12, 13). SRH is also deemed as a comprehensive indicator of a person's health, as it is related to health behaviors, well-being, changes in health over time, socio-economic conditions, and overall quality of life (14–17).

### Health Challenges Among Immigrants

Migration is a life event that can impact the health and well-being of individuals (18–21) and immigrants often tend to experience poorer health compared with the general population (22–26). The causes of poorer health among immigrants are complex and need to be seen under the prism of socio-ecological perspectives (27). Social determinants of health, health-related behaviors, and the acculturation process contribute to the immigrant's health status (28–30). According to the “healthy immigrant effect,” those leaving their country of origin are usually younger, healthier, and highly resourceful (31, 32). For this reason, immigrants, upon their arrival in the new country, tend to be healthier than the host population. However, their health tends to deteriorate after some time. Although several studies support this hypothesis (33–36), others provide contrasting evidence (24, 37–40). Determinants going beyond individual characteristics are claimed to be more relevant in explaining differences in health (41). Theories related to discrimination and structural racism point to the impact of entitlements, opportunities, and expectations meeting immigrants in a new country. Integration policies in European countries can influence the health outcomes of immigrant populations (42–44). Immigrants living in more inclusive multicultural countries were found to have better health than those living in exclusionist countries (45, 46). The capacity, or opportunity for immigrants in integrating into new societies, often referred to as acculturation, has also been used for understanding health among immigrant groups (29, 47).

To understand the nexus between migration and health, a growing number of studies suggest the importance of looking at the whole migration process, including where immigrants come from, why they move, how they moved, and their living conditions in the country of resettlement (48, 49). Coming from countries with a good economy and an efficient health system and moving to a country offering universal entitlements to health care, may have a different impact on health than escaping war or famine, embarking on a long perilous journey, and living in uncertain conditions (21). Acknowledging that the experience of migration can be different in different migration groups, the literature tends to categorize immigrants into asylum seekers, refugees, forced migrants, labor migrants, expats, “Erasmus generation” and new mobilities (48, 50–52). While the first often find themselves at the border of societies, the last ones are more often part of a mobile generation in a world of globalized opportunities. This way of categorizing and defining the flux of people moving outside the boundaries of their own country synthesizes important information about the resources and opportunities these different groups have that may influence their health.

### Health in the Context of Intra-European Migration: The Case of Italian Immigrants

The existing studies on the health challenges among immigrant populations in European countries mostly rely on studies conducted among immigrants from outside Europe (10, 25) and few studies investigate health in the context of intra-European migration (38, 40, 53, 54). A case of intra-European mobility is the one represented by Italians moving to Norway (55). Italians' immigration to Norway, although still relatively low in number, has been steadily increasing since the establishment of the EEA Agreement in 1994, and it has tripled since the economic crisis of 2008 (56). According to the Registry of Italians Residing Abroad (AIRE) for 2020, the Italians residing in Norway were 7,885 of these, 43% are women and 57% are men (57). Norway as an attractive country of migration for Italians is a rather new phenomenon. However, the immigration of Italians to Norway has most likely existed since the late eighteenth and early nineteenth centuries (58). The nineteenth-century emigration to Norway had its roots in a past of territorial mobility with a strong mercantile and artisanal component (59). The largest group of Italians in Norway was made up of itinerant sellers of chalk and iron objects, itinerant musicians, and, to a lesser extent, ice cream makers. A small number of people were employed in other occupations, such as traders, sailors embarked on the Norwegian naval fleet, and miners (55).

There are no data attesting the sanitary or health conditions of Italian emigrants in Norway at that time, but sources are documenting where and how they lived. From this information, it is possible to hypothesize that their living conditions were not ideal, as Italian immigrants often had to share their living space with 20–30 people in extremely cramped spaces in a perennial state of overcrowding (58, 59). In Norway, any form of assistance to Italian immigrants was made even more difficult by the absence of a diplomatic representation (55). The country until 1905 was in fact in union with Sweden, which implied legislative autonomy in domestic politics, but dependence on foreign policy. Hence the absence of Italian diplomatic representations in the Norwegian territory (55).

After the Second World War, Norway, like many other European countries, including Italy, experienced an economic phase of full expansion which increased the need for labor in the country, stimulating the arrival of new groups of foreigners (55). During this period, important changes were made to the immigration law, which became relatively more liberal than the one previously in force, enacted in 1927. The 1956 reform allowed foreign workers to enter and settle in Norway with more ease (60). The Italian immigrants who arrived in Norway in the 1950s were mainly young male workers, single or heads of household, generally with a low level of education (55). The low level of education was not strictly found amongst migrants but reflected the general Italian society of that historical period where the level of education was on average low. In Italy in 1961, according to Istat data, only 1.7% of the population had a degree and just over 5% had a diploma (55). Despite the ease with which one could enter the country and the need for manpower, very few Italians chose Norway in the immediate postwar period. The country, in this particular period, was not particularly attractive in the eyes of foreign workers and Norway had not carried out any recruitment policy through bilateral agreements between states, like other European countries, such as Germany or Belgium. This lack of agreements between states meant that the Italians who arrived in Norway in that period did so independently, by their own choice, because they had a relative, a friend, someone who had been there in the past or for love (55).

Compared to the modest figures that have characterized the immigration of Italians to Norway in the past, since 2008 the number of those who moved to this country has experienced an unprecedented surge (56). This wave of migration can be a part of new forms of mobility (61, 62). Research shows that among today's Italian immigrants in Norway—contrary to what happened in the past- there is a high percentage of highly educated and skilled young people moving for study or opportunities for qualified jobs (56). What emerges is, therefore, a different kind of immigration than in the past, more educated, more technological, more cosmopolitan, and more mobile (63). Contemporary Italian mobility to Norway has also seen a considerable increase in the female component and of women that move alone (55).

### Aims of the Study

As studies of migrants' health in the context of intra-European migration are few, investigating this phenomenon can provide new knowledge on the specific contribution of migration as a determinant of health: what happens to the health of a group of immigrants with relatively high resources when they move? This topic will be addressed in this study by investigating the case of Italian immigrants in Norway, a group that remains largely under-researched despite its growth. More specifically, the aim of this study was three-fold: (i) examine the levels of SRH among Italian immigrants in Norway as compared with the Norwegian population and the Italian population in Italy; (ii) examine the extent to which the Italian immigrant perceived that moving to Norway had a positive or negative impact on their SRH; and (iii) identify the most important factors predicting SRH among Italian immigrants in Norway.

## Methods

The study is part of a research project on the health among Italians living in Norway “*Mens sana in corpore sano*,” conducted in collaboration with the Oslo's Committee of Italians living abroad (Comites) and the Italian Embassy in Norway (56). A cross-sectional survey was conducted between 15 March and 24 April 2019 (hence, before the lockdown due to the COVID-19 pandemic) among adult Italian immigrants living in Norway (56). Since an updated contact list of all the Italians living in Norway was not available, the survey was distributed through different channels, including invitations through the Comites' mail-list and announcements on the Italian Embassy's website and different online groups for Italians living in Norway. The inclusion criteria were: being an Italian-speaking immigrant residing in Norway, age 18 years or older, and having spent most of one's childhood (up to age 16 years) in Italy. Compliance with these criteria was assessed through control questions in the survey. A total of 330 people responded to the survey, of which 321 met all inclusion criteria. A comparison of sociodemographic variables (gender, age, educational level, and region of residence) of the sample with figures provided by national registers, such as the Registry of Italian Citizens Residing Abroad (AIRE), revealed that our sample had a larger proportion of women, mid-aged individuals, people with a higher educational level, and people living in the region of Oslo-Akershus. To enhance the sample's representativeness, the original dataset was oversampled to match the proportion of key sociodemographic characteristics of the reference population (Italian residents in Norway according to AIRE's data) using the ADASYN method (64). More specifically, the oversampling was based on the expected distribution of age and education level, which also resulted in an acceptable adjustment of gender and place of residence (final *n* = 531). More details about the oversampling procedures, including a comparisons of key socio-demographic characteristics of the reference population compared to the original sample and the resampled dataset, are described in Supplementary Materials and in a previous publication from the same study (65).

### Instruments

The questionnaire used in the *Mens Sana in Corpore Sano* study (which was in the Italian language) was developed to allow comparisons with existing surveys in Norway and Europe. More specifically, the items were taken from or closely inspired by items used in Survey on living conditions (9) and the Eurostat Population and social conditions (8).

#### Self-Rated Health (Main Outcome)

The main outcome was SRH, which was assessed through a single item asking “In general, how would you rate your health?” (1 = “Very bad”, 2 = “Bad”, 3 = “Neither good nor bad”, 4 = “Good”, 5 = “Very good”). For the analyses, the item was dichotomized into two levels in agreement with the approach used by Statistics Norway (9). Eurostat (8), as well as other studies on the Italian population (6): ‘worse SRH' (which included the response options “Very bad”, “Bad”, and “Neither good nor bad”) and ‘better SRH' (including the response options “Good” and “Very good”).

#### Perceived Impact of Moving (Secondary Outcome)

A variable assessing the Italians' perceived impact of migration to Norway on their SRH was used as an indication of the extent to which, all in all, the respondents perceived that their health was influenced by the migration process. This was measured with a single item inquiring the following: “Imagine that you did not move to Norway and, instead, continued to live in Italy. What of the following statements would better reflect your health in such a hypothetical circumstance?” The response options were: 1 = “My health would have been better in Italy than now in Norway”, 2 = “My health would have been more or less the same in Italy as it is now in Norway”, 3 = “My health would have been worse in Italy than it is now in Norway”, and 4 = “I don't know.” The item was presented immediately after the SRH instrument, to prompt respondents to refer to the same understanding of “health” as in the SRH measurement.

#### Predictors of SRH

The *Mens Sana in Corpore Sano* survey included a large number of items relative to the Italian immigrants' life in Norway (56). After a preliminary screening of the dataset, some items were excluded because considered not relevant for this specific study (e.g., specific information about food habits or physical activity practice), while other items were re-coded to better fit the purpose of the study (e.g., response options with a small number of responses were merged and the level of some categorical variables was re-coded to facilitate interpretation of the findings). Items such as Satisfaction with Life and Perceived moving on Health were excluded as predictors because of potential endogeneity problems. Eventually, 35 variables were included in the machine learning analysis as predictors of SRH. The list of the included variables and a short description are provided in Table 1, while a more detailed description of all variables is presented in Supplementary Material.

**Table 1:** Predictors of Self-rated health included in the machine learning analysis.

**Variables relative to sociodemographic characteristics**	
**Gender:**	**Male or female**
**Age:**	**Years**
**Educational level**	**Highest completed educational title (Primary or lower, High school, B.A. degree, M.A. degree or equivalent, Doctoral degree)**
**Region of residence**	**Whether or not the respondent lived in Oslo/Akershus (i.e., the most urbanized and densely populated region of Norway)**
**Living alone**	**Whether or not the respondent lived alone**
**Living with children**	**Whether or not the respondent lived with children, own or of the partner's**
**Living with partner**	**Whether or not the respondent lived with a partner**
**Occupational situation**	**The respondent's occupation at the moment of the survey (unemployed, student, engaged in occasional occupation, self-employed, hired by the piece, employed with a term contract, employed with a permanent contract, other)**.
**Satisfaction with occupation**	**The extent to which the respondent perceived that their occupation was adequate with respect to their educational background (1 = “My current occupation is unsatisfactory considering”, 4 = “Considering my educational background, I am highly satisfied with my occupation)**.
**Variables relative to interaction with the health system**	
**Trust in the Norwegian Health System**	**The extent to which the respondents expressed their trust in the Norwegian health system, including medical staff and other health personnel (0–10 scale)**.
**Health literacy**	**Short Version of the European Health Literacy Survey Questionnaire (HLS-Q12 assessing the participants beliefs relative to their ability to find and understand information relative to their own health (1 = “Very difficult”, 4 = “Very easy”; α = 0.83). The resulting total score ranges between 12 and 48, with ratings below 26 indicating “inadequate” health literacy, ratings between 27 and 32 indicating “marginal” health literacy, ratings between 33 and 38 indicating ‘intermediate' health literacy, and ratings above 39 indicating “advanced” health literacy**.
**Empowerment relative to communication**	**One item assessing the participants' belief relative to their ability to actively participate in the dialogue with health personnel (1 = “Very difficult”, 4 = “Very easy”)**.
**Empowerment relative to health behaviors**	**Three items assessing the participants' beliefs relative to their ability to take control of their own healh in daily life by implementing health goals and health-related behaviors (1 = “Very difficult”; 4 = “Very easy”; α = 0.75)**.
**Variables relative to health-related behaviors and social relations**	
**Food habits**	**The extent to which the respondents perceived that, all in all, their food habits were healthy (1 = “Not at all”; 5 = “Absolutely agree”)**.
**Weekly physical activity**	**Whether the respondent reported to engage in health enhancing physical activity broadly in line with the WHO's definitionfour levels: never, <2.5 h/week, between 2.5 and 5 h/week, 5 or more h/week**
**Tobacco usage**	**Whether the respondents daily, occasionally, or never made use of tobacco**
**Nature restoration**	**The respondent's perceived opportunities to engage in restorative nature experiences (1 = “Not at all”; 4 = “Absolutely agree”)**
**Contact with good friends**	**The frequency of spending time with people they value as good friends (1 = “Never/less than once a year”, 6 = “Almost every day”)**
**Contacts with Italian relatives**	**The frequency of having contacts with their Italian family of origin (1 = “Never/less than once a year”, 6 = “Almost every day”)**
**Trust in people**	**The extent to which the respondents expressed their trust in people, in general (0–10 scale)**.
**Perceived impact of moving on health-related behavior and social relations**	
**Perceived impact of moving on food habits**	**The extent to which the respondents perceived that their food habits were positively or negatively influenced by the migration process**.
**Perceived impact of moving on physical activity habits**	**The extent to which the respondents perceived that their physical activity was positively or negatively influenced by the migration process**.
**Perceived impact of moving on social relationship:**	**The extent to which the respondents perceived that their social life was positively or negatively influenced by the migration process**
**Variables relative to indicators of acculturation**	
**Years of permanence in Norway:**	**Number of years since moving**
**Language proficiency**	**The respondents' perceived proficiency in the Norwegian language (from 1 = “very poor”, to 5: “very good”)**.
**Identifying as a Norwegian**	**The extent to which the respondents identified completely, partially, or not at all as a Norwegian**.
**Identifying as an immigrant**	**The extent to which the respondents identified completely, partially, or not at all with the status of immigrant**
**Italian friends**	**Whether the respondents have none, less than half, about half, more than half, or only friends of Italian origin**.
**Belief about future**	**The respondents' belief regarding their future place of residence, either they intended to move again (back to Italy or to another country) or continue living in Norway**.
**Variables relative to factors associated with the migration**	
**Family-related reasons for moving**	**Whether or not the participant had family-related reasons for moving to Norway (i.e., following a partner, family member/s, or re-uniting with partner or other family member/s)**
**Job-related reasons for moving**	**Whether or not the participant had job-related reasons for moving to Norway (i.e., having received a job offer or looking for job opportunities)**
**Other reasons for moving:**	**Whether or not the participant had any other reasons for moving to Norway**
**Moved alone**	**Whether or not the respondent moved to Norway alone**
**Moved with family**	**Whether or not the respondent moved to Norway with their family or part of it (either they traveled together or moved to reunite with the partner of family member/s)**
**Moved with others**	**Whether or not the respondent moved to Norway with anyone other than partner or family**.


## Analysis

### Descriptive Statistics

Patterns of association among the independent variables were preliminarily examined through visualizations in a correlation matrix and a correlation network. Descriptive statistics for SRH, perceived impact on SRH, and all other variables were performed and presented as percentages (%), Median and inter-quartile range (Q1-Q3), or means (M) and standard deviations (SD).

#### Comparison of SRH of the Italian Immigrants With the Norwegian and Italian Population

A Chi-squared analysis was performed to compare the Italian immigrants' SRH with figures about the Norwegian population according to aggregated data retrieved from Eurostat's SRH statistics, which is part of the Health in the European Union survey (8). For this comparison, the dichotomous version of SRH (two levels: ‘worse' and ‘better' SRH, recoded as described in the instruments section) was used.

#### Machine Learning Approach Predicting SRH Among the Italian Immigrants

Machine learning is an application of artificial intelligence that automatically detects multidimensional and non-linear patterns within a high-volume dataset to make predictions based on these patterns (66). Since this methodology can be applied to all types of data, it is nowadays widely used in a large number of research fields, including health (67). For example, machine learning techniques have been previously used to investigate the predictors and correlates of SRH based on survey data [see e.g., (68, 69)] as well as other established public health indicators, such as mortality risk, in different contexts and populations [see e.g., (70–72)]. Machine learning approaches are particularly well-suited when the goal is to produce a high-precision predictive model, inductively identifying the most relevant predictors from a large set of data; this differs from traditional statistical models, which are often seen as more suited when the goal is to deductively make inferences about the relationship of specific variables. Hence, given our purpose of identifying the most important factors predicting SRH among Italian immigrants in Norway, a machine learning approach was deemed particularly appropriate.

In this study, a framework of data analytics was used to identify the most important correlates of SRH among Italian immigrants. Five machine-learning models Decision Tree Classifier (DTC); Random Forest Classifier (RFC); Logistic Regression Classifier (LR); eXtreme Gradient Boosting classifier (XGB); Adaptive Boosting classifier (ADA) were trained to predict both SRH by evaluating 35 independent variables (see section Instruments). A 10-folds cross-validation approach was used to test the accuracy of these machine-learning models (73). Moreover, to validate the prediction ability of these models, a dummy classifier that randomly predict the output in accordance with the dependent data distribution was cross-validated to check the real prediction ability of the machine-learning models. Similar or higher results of dummy classifier compared to “real” models indicate that they were not able to detect patterns in data that permit to accurately predict the output. The model goodness was assessed by four metrics: (i) precision (i.e., the ratio of correctly predicted positive observations to the total predicted positive observations); (ii) recall (i.e., the ratio of correctly predicted positive observations to all observations in actual class); (iii) f1-score (i.e., the weighted average of Precision and Recall); (iv) accuracy (i.e., the ratio of correctly predicted observation to the total observations) (73). To globally and locally explain the decision-making process of the models, SHapley Additive exPlanations (SHAP) values were computed to explore the relationships between variables for predicted cases. In particular, SHAP assigns to each variable an importance value for a particular prediction (based on a linear function) permitting an evaluation of the influence of each variable on the final prediction. In particular, the collective SHAP values can show the extent to which each predictor contributes, either positively or negatively, to the target variable (74).

## Results

### Sample's Description

Descriptive statistics for SRH, the perceived impact of moving, and all variables included in the predictive model are presented in Table 2. The mean age of the Italian immigrants was 40.54 years (SD = 10.78 years). The proportion of men was sensibly larger than the women's (60.45% and 39.55%, respectively). In general, the Italian immigrants tended to have a higher educational degree, with 61.39% having achieved a university degree, either at Bachelor (10.73%), Master (33.33%), or Doctoral level (17.33%). This represents a noteworthy high educational level. The majority lived in the most central and urbanized region of Norway (Oslo and Akershus, 56.50%). Around 70% moved to Norway alone. The main reason for moving was work (43%), followed by family reasons (25%) and other reasons, which mainly consist of study reasons (15%). The large majority reported having a relatively stable occupational situation, with either a term- or a permanent contract (26.18 and 47.08%, respectively). The 5% indicated that were unemployed. The mean health literacy ratings were 35.34 ± 3.91, indicating that, on average, the Italian immigrants had intermediate levels of health literacy. More specifically, the large majority of the Italian immigrants (81%) reported ratings indicative of ‘intermediate' health literacy, with a relatively small proportion reporting ‘inadequate' (3%) or ‘marginal' (7%) health literacy, while 9% reported an ‘advanced' health literacy.

**Table 2:** Descriptive statistics of Self-rated health, perceived impact of moving, and other information about the health and living conditions of Italian immigrants in Norway (oversampled dataset, *n* = 531).

**Variable**	**Descriptive** **statistics**
***Sociodemographic characteristics***	
Age, M ± SD	40.54 ± 10.78
Gender, %	
Man	60.45%
Woman	39.55%
Educational level, %	
Primary or lower	4.71%
High school	33.90%
B.A. degree	10.73%
M.A. degree or equivalent	33.33%
Doctoral degree	17.33%
Region of residence, %	
All other regions	43.50%
Oslo & Akeshus	56.50%
Occupational situation, %	
Unemployed	4.90%
Student	2.64%
Occasional occupation	2.45%
Self-employed	7.16%
Hired by the piece	7.34%
Term contract	26.18%
Permanent contract	47.08%
Other	2.26%
Satisfaction with occupation %	
Unemployed	6.09%
Unsatisfactory	14.00%
Satisfactory but not consistent with educational background	19.68%
Satisfactory and consistent with educational background	36.51%
Highly satisfied with occupation	23.73%
***Interaction with the health system***	
Empowerment relative to health behaviors, Median [Q1–3] (1–4 scale)	3 (3)
Empowerment relative to communication, M ± SD (1–4 scale)	3 [3]
Health literacy, M ± SD (total score)	35.34 ± 3.91
Trust in the health system, Median [Q1–3] (1–10 scale)	6 [4–7]
***Health related behaviors and social relations***	
Healthy food habits, Median [Q1–3] (1–4 scale)	3 [2–4]
Nature restoration, Median [Q1–3] (1–4 scale)	2 [1–4]
Tobacco usage, %	
No	79.47%
Yes, occasionally	13.37%
Yes, regularly	7.16%
Weekly physical activity, %	
No regular physical activity	23.16%
<2.5 h/week	23.16%
Between 2.5 and 5 h/week	33.33%
5 h/week or more	20.34%
Contacts with Italian relatives, %	
Never/ < once a year	0.75%
About once a year	1.32%
Some times a year	5.84%
About once a month	11.68%
About once a week	47.27%
Almost every day	33.15%
Contact with good friends, %	
Never/ < once a year	1.32%
About once a year	2.45%
Some times a year	22.98%
About once a month	27.31%
About once a week	36.53%
Almost every day	9.42%
Living alone, % “Yes”	41.30%
Living with children, % “Yes”	25.24%
Living with partner, % “Yes”	67.32%
Trust in people, Median [Q1–3] (1–10 scale)	5 [6, 7]
***Perceived impact of migration***	
Perceived impact on food habit, %	
I don't know	4.12%
Negative impact	51.37%
No impact	36.08%
Positive impact	8.43%
Perceived impact on physical activity, %	
I don't know	0%
Negative impact	22.04%
No impact	37.76%
Positive impact	40.20%
Perceived impact on social relationships, %	
I don't know	2.53%
Negative impact	72.6%
No impact	21.21%
Positive impact	3.31%
***Indicators of acculturation***	
Belief about future^a^, %	
Live in Norway for a short period	6.03%
Move back to Italy when I'll be old	31.45%
Move in some other country	23.73%
Spend the rest of my life in Norway	25.24%
I don't know	13.56%
Identifying as an immigrant, %	
I don't know	2.32%
No	18.34%
In part	48.26%
Yes	31.08%
Identifying as a Norwegian, %	
I always identify as an Italian	54.42%
I predominantly identify as an Italian	37.15%
I identify as Italian and a Norwegian in equal extents	8.03%
I predominantly identify as a Norwegian	0.40%
I always identify as a Norwegian	0.00%
Italian friends, %	
None	18.64%
< half	38.23%
About half	19.96%
>half	20.34%
All of them	2.82%
Language proficiency, %	
Very poor	13.37%
Poor	23.16%
Intermediate	27.68%
Good	22.79%
Very good	12.99%
Years of permanence in Norway, M ± SD	8.75 ± 7.78
***Factors associated with the migration***	
Moved alone, % “Yes”	69.63%
Moved with family, % “Yes”	29.38%
Moved with others, % “Yes”	1.69%
Moved for family-related reasons, % “Yes”	26.74%
Moved for job-related reasons, % “Yes”	43.31%
Moved for other reasons, % “Yes”	15.63%
***Self reported health***	
Worse (total)	30.89%
Very bad	0.38%
Bad	6.78%
Neither bad, not good	23.73%
Better (total)	69.11%
Good	57.06%
Very good	12.05%
Perceived impact of migration on SRH, %	
I don't know	6.10%
Negative impact	22.76%
No impact	55.08%
Positive impact	16.06%

*[a] Belief about the future is here presented as it was assessed in the questionnaire, but it was dichotomize for the purpose of the Machine learning analysis (0 = “Plan to move again/I don't know” and 1 = “Spend the rest of one's life in Norway”) (oversampled dataset, n = 531)*.

### SRH Compared With the Norwegian and Italian Population

**Figure 1:**
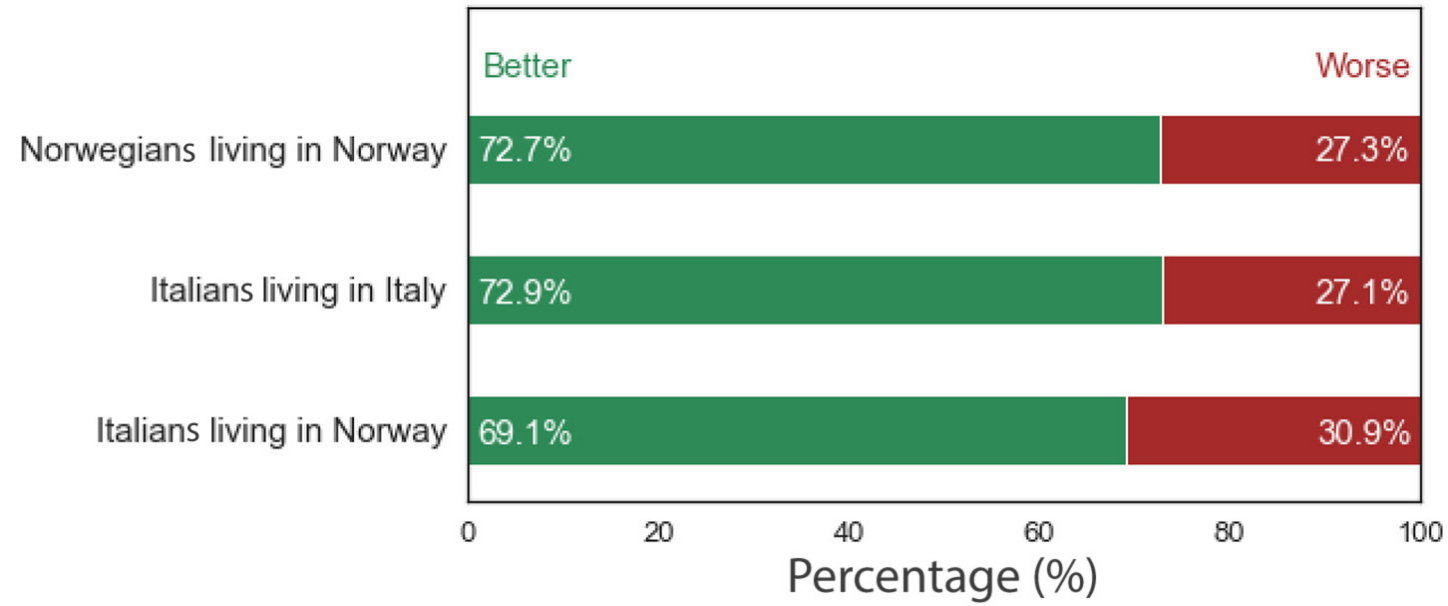
Self-rated health among Norwegians, Italians, and Italian immigrants living in Norway.

Most of the respondents (69.11%) reported “better” SHR levels (i.e., rated their health as “good” or “very good”) while 30.89% reported worse SHR levels (rated their health as “very bad”, “bad”, or “neither bad not good”). These proportions were not significantly different compared with figures for the Norwegian population (72.7% reporting better SRH levels; χ^2^ = 0.65, *p* = 0.42), nor compared to the Italians living in Italy (72.9% reporting better SRH levels; χ^2^ = 0.81, *p* = 0.37). Figure 1 illustrates the proportion of worse' and ‘better' SRH levels among the Italian immigrants in Norway, the Norwegian population, and the Italian population.

### Perceived Impact of Moving

Looking at the variable Perceived impact of moving, it emerges that the majority of Italian immigrants (55.08%) perceived that their health would have been more or less the same if they continued living in Italy, with 16.06% perceiving their health was better in Norway compared to how it would have been if they continued living in Italy. The prevalence of Italian immigrants perceiving that their health was worse in Norway compared to what would have been if they continued living in Italy was 22.76%. The remaining 6.10% selected the response option “I don't know”.

### Variables Predicting SRH

Table 3 shows that among the machine learning models tested, the Random Forest Classifier (RFC) most accurately predicted whether the Italians living in Norway reported ‘worse' or ‘better' SRH levels (accuracy = 83.07 ± 2.66%). The higher prediction ability of the machine learning models compared to the Dummy one (accuracy = 56.13 ± 2.67%) corroborate the fact that these models could detect patterns in data that permit to accurately distinguish between individuals with different perception SRH levels.

**Table 3:** Accuracy of the machine learning models predicting Self-rated health (SRH).

**Model**	Precision*	Recall*	F1-score*	**Accuracy**
DTC	75.05 ± 2.08	74.44 ± 2.31	74.61 ± 2.14	74.44 ± 2.31
RFC^a^	83.04 ± 2.74	83.06 ± 2.66	82.04 ± 3.10	83.07 ± 2.66
LR	69.31 ± 2.50	71.31 ± 2.13	69.31 ± 2.45	71.31 ± 2.13
XGB	78.95 ± 2.61	79.50 ± 2.26	78.84 ± 2.69	79.50 ± 2.26
ADA	74.28 ± 2.30	75.19 ± 2.19	74.18 ± 2.35	75.19 ± 2.19
Dummy	56.18 ± 1.59	56.13 ± 2.67	56.07 ± 2.03	56.13 ± 2.67

*Metrics referring to the weighted values about the two classes.

a *Model with the highest prediction goodness*.

*DTC, Decision Tree Classifier; RFC, Random Forest Classifier; LR, Logistic Regression; XGB, eXtreme Gradient Boosting classifier; ADA, Adaptive Boosting classifier*.

**Figure 2:**
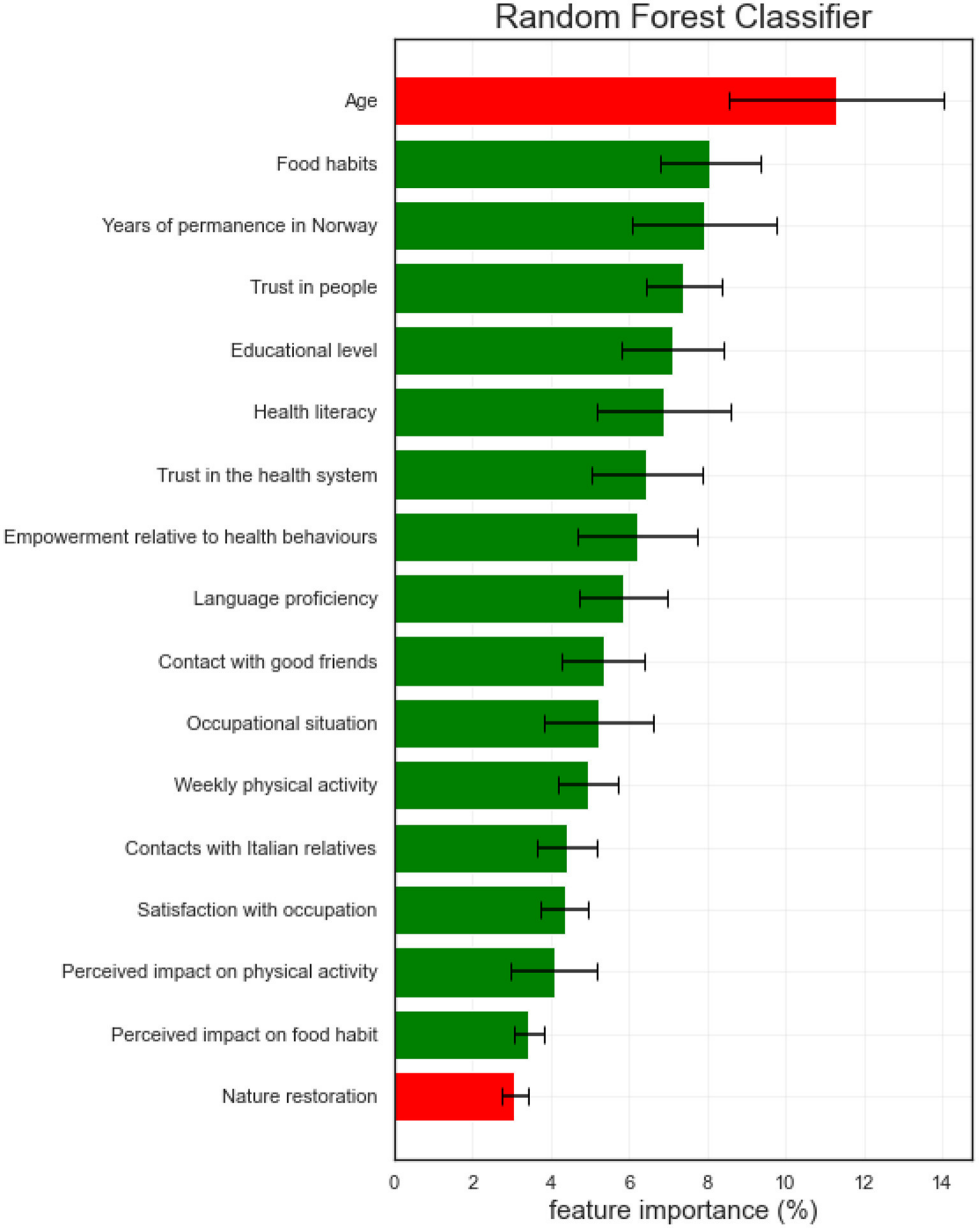
Importance (expressed as SHAP values) of the relevant variables predicting SRH among Italian immigrants in Norway, according to the RFC model. Red bars refer to the negative influence of the independent variables on the dependent one, while the green one refers to a positive influence.

Figure 2 presents the importance (SHAP values), expressed as a percentage (%) and 95% confidence interval (95% C.I.), of the variables identified by the RFC model to predict ‘worse' or ‘better' SRH among the Italian immigrants. Of the 35 variables included, the RFC model identified 17 variables as relevant in predicting SRH among the Italian immigrants. Among these, Age was the most important [11.29% (8.54%, 14.04%)], which was negatively associated with SRH, indicating a greater likelihood of reporting worse SRH with increasing age. The second most important predictor of SRH was Food habits [8.07% (6.78%, 9.36%)], with those reporting to have more healthy food habits being more likely to also report better SRH. Years of permanence in Norway was the third most important predictor of SRH [7.90% (6.05%, 9.75%)], with an increased likelihood of reporting better SRH among those who lived for a longer time in Norway. The fourth most important predictor of SRH was Trust in people, with those reporting to have more trust in people being more likely to also report better SRH. Educational level [7.12% (5.80%, 8.43%)] and Health literacy [6.87% (5.18%, 8.57%)] were the fifth and sixths, respectively, most important predictors, indicating that those who have higher education attainment and greater health literacy were more likely to report better SRH. These were followed by two variables indicative of the Italians' interaction with the health system, namely Trust in the health system [6.45% (5.02%, 7.88%)] and Empowerment relative to health behaviors [6.20% (4.67%, 7.73%)]. Higher Norwegian language proficiency [5.84% (4.72%, 6.96%)], more frequent Contact with good friends [5.34% (4.28%, 6.40%)], having a more stable occupational situation [5.23% (3.85%, 6.62%)], higher levels of weekly physical activity [4.96% (4.20%, 5.73%)], more frequent Contact with Italian relatives [4.42% (.66%, 5.17%)], and higher levels of satisfaction with occupation [4.35% (3.76%, 4.94%)] were also all positively associated with better SRH. Similarly, the Perceived impact of moving on one's physical activity [4.08% (2.98%, 5.18%)] and food habits [3.44% (3.06%, 3.80%)] showed positive associations, indicating that those who perceived that these health-related behavior improved as a result of moving to Norway were more likely to report better SRH. Finally, the frequency of Nature restoration was negatively associated with SRH [3.07% (2.73%, 3.04%)], indicating that those who frequently experience nature's quietness were more likely to report worse SRH.

## Discussion

### Summary of Main Findings

This study aimed to investigate (i) SRH among Italian immigrants living in Norway as compared with the Norwegian and Italian general population, (ii) the extent to which they perceived that their SRH was influenced positively or negatively as a result of moving, and (iii) the factors predicting better or worse SRH among the Italian immigrants in Norway. A large majority reported experiencing good or very good health. The study found no statistically significant difference in SHR ratings among the Italian immigrants as compared with the Norwegian and Italian general population. Most of the Italian immigrants perceived that their SRH was more or less the same in Norway as it would have been if they continued living in Italy, though a relatively large group perceived a negative impact on their health. Several factors contribute to explaining ‘worse' or ‘better' SRH levels. Sociodemographic characteristics such as age and education were among the variables that most influenced SRH. Gender, somehow surprisingly, did not emerge as a relevant factor predicting SRH. Health-related behaviors (primarily food habits) were also relevant predictors of SRH. Indicators of acculturation (most of all Years of residence in Norway, followed by language proficiency), and health-related factors (especially Health literacy) also emerged as important predictors. Variable related to trust, such as trust in people and trust in the health system were also of high relevance.

### Better or Worse Health?

Our study indicates that most of Italian immigrants in Norway generally perceive having good health, and similar SRH compared to the Norwegian population. This is an important finding as other studies among Italian immigrants living in other countries than Norway suggest that the SRH of immigrants is generally worse than the host population's (25, 38, 40). Importantly, Italians in Norway reported similar SRH also when compared with Italians living in Italy. This, alongside the presence of a consistent group of Italians stating they would have similar health if they continued living in Italy, corroborate the finding that moving to Norway did not have a major impact on the health of the Italian immigrants. The differences with SRH of Italian immigrants in other countries may be due to the characteristics of the Italian immigrant population in Norway. The Italians in the overmentioned studies were older, with lower educational levels, and had poorer working conditions than those in our sample (38, 40).

As education is a strong predictor of health and health-related behaviors, when considering our findings it is important to bear in mind that the Italians participating in the study had a particularly high educational level, with more than 40% having a University degree and almost one-fourth a Ph.D. By way of comparison, in 2019, the rate of Italians aged 25–64 with a university degree was 19.6% (75), while in Norway those with a university degree were 33.6% and 1% with a Ph.D. (76). Previous studies have shown that people with high educational levels tend to rate their health more positively than those with lower education (15, 77). This is corroborated by the fact that, in our predictive model, educational level was one of the most relevant factors predicting better levels of SRH among Italian immigrants. Given the high level of education among Italians living in Norway, one may therefore expect better levels of SRH among the respondents in our study when compared to the Norwegian population, as well as the Italians living in Italy. The fact that this did not occur may suggest that the positive impact on health of being highly educated may be weakened by other factors throughout the migration process and resettlement in a new country.

The absence of a significant impact of gender in SRH is also an interesting finding. Several studies show the presence of a gender gap in SRH, with women generally perceiving worse health than men (78, 79). Figures from the European Health Survey indicate the presence of such differences in SRH between women and men in Italy, and to a much lesser extent in Norway (8). In the present study, the lack of a significant gender gap may be explained by the fact that the Italian immigrant women in Norway tend to be younger and more highly educated compared to their male counterparts (as shown by the moderate bivariate correlation of gender with educational level and age in Supplementary Figure 1). The masking of a statistically significant gender differences, when measures of socioeconomic status are considered, emerged also in other studies (79).

### The Healthy Immigrant Hypothesis

Age emerges as the strongest (negative) predictor of SRH. This is not surprising, and it is in accordance with previous studies showing that SRH tends to worsen with age (78, 80). This finding becomes however interesting when we consider that, in our study, years of permanence in Norway emerged, instead, as a positive predictor of SRH. Preliminary exploration of the data (see Supplementary Figure 1) showed that age and years of permanence in Norway were strongly correlated and positively with each other, meaning that most of the older immigrants had lived in Norway for a longer time. This is probably explained by the fact that Italian immigrants tend to arrive in Norway as young adults. The finding that SRH increases the longer one lives in the host country is in contrast with other studies and, in particular, with the “healthy immigrant hypothesis”, stating that health tends to deteriorate with time spent in the new country (21, 81). One possible explanation is that previous studies considering the impact of acculturation on health have focused on migration from lower to higher-income countries or on forced migrants (25, 34, 82). Adoption of lifestyles common in western countries such as a more sedentary life and higher consumption of processed food is often associated with deterioration in health (27). Other studies have also indicated that immigrants from lower-income countries tend to have working conditions that negatively affect their health (28, 82). These conditions may not be the case for the Italian immigrants in our study who, coming from western countries themselves, are likely to be already acculturated into western lifestyle patterns. In addition, they may have better working conditions than other immigrant groups.

Years of permanence, together with language proficiency (which also emerged as a significant and positive predictor of SRH in this study), are commonly used as indicators for measuring acculturation, defined as taking on values and practices of the host country (83). Higher language proficiency can be of relevance for participation in society, and feelings of mastering everyday tasks, thus contributing to a positive perception of own health (47).

### Trust and Social Relations as a Determinant of Health

“Trust in people” is an indicator of social capital and cohesion (84). Several studies have indicated that trust is associated with health and wellbeing (85, 86). The association between trust and health can be related to the fact that trust reduces stress and may promote involvement in social networks, which themselves improve health (87). Trust can also be seen as a way of managing uncertainty in life, which can be even more important when resettling and integrating into a new country (88). Migration is often regarded as a life event that may lead to stressful situations (89, 90) as people need to know and adapt to different norms and ways of living and may experience discrimination, and weaker social relations (91, 92), It is, therefore, an important finding of our study that trust emerged as a relevant predictor of SHR among Italian immigrants.

Together with trust in people, also trust in the health care system has been regarded as important for SHR. Trust in the health system plays a role in explaining one's access to and utilization of medical care, adherence to medications, continuity of care, and -thus- SRH (93). Norway is a country that in several studies has scored high in trust in the institutions among the general population (94). However, distrust in the health care system has been expressed in several studies among immigrant populations coming from not western countries due to discrimination and stigmatization that immigrants may have experienced (95, 96). This is consistent with the findings of our study, indicating relatively low levels of trust in the health system among Italian immigrants. Given the importance of trust in the health system for SRH, promoting trust in the health care system needs to receive more attention as a health promotion campaign among immigrants, including those coming from EEA countries, such as the Italians.

Social relations are an important determinant of health (30, 97). Also in our study having regular contact with good friends and with Italian relatives is positively associated with good SRH. It is interesting to note that the variable social relation did not significantly affect SRH in our model. In a previous article from the same study (55) including qualitative interviews, Italian voiced in fact difficulties in establishing friendships relation with Norwegians. One possible reason can be the increased facility that immigrants have in maintaining meaningful relations with family and friends in a digital world (98), so when answering the question related to social relations they may have referred to social relations at large (both in Norway and in Italy).

### Health Literacy, Empowerment, and Health-Related Behaviors

Health literacy together with empowerment relative to health behaviors emerged as highly relevant predictors of SRH. Other health-related behaviors such as food habits and weekly physical activity were also identified as factors supporting better SRH. Altogether, these findings are consistent with other studies documenting the relevance of health literacy and health-related behaviors for SRH (99, 100).

Health literacy has been defined as people's knowledge, motivation, and competencies to access, understand, appraise and apply health information to make judgments and take decisions in everyday life concerning health and utilization of professional health services (101). Preliminary findings on the health literacy among Italian immigrants in Norway have been presented in a previous publication from the same study, indicating that the Italian immigrants tend to report slightly (but significantly) lower levels of health literacy compared with the general Norwegian population (102). Previous studies have shown a strong correlation between health literacy and education (103, 104). Given the high level of education of our sample, considerations concerning this issue are therefore warranted. This finding may be explained by the fact that, in the context of migration, even people with high health literacy in their own country (as we could expect given the high educational level of the sample) may find themselves having lower health literacy in the country of resettlement, due to language barriers and lack knowledge of the health care system (105).

The only finding that was in contrast with current scientific literature was the negative association of SRH with the Italian immigrants' frequency of nature experiences. In the past decade, a large body of evidence has demonstrated that contact with nature provides a wide range of health benefits. WHO, for instance, has reviewed the evidence on the health benefits of urban nature (parks, green corridors, residential greenery, etc.), concluding that these spaces significantly contribute to reducing morbidity and mortality among urban residents by eliciting stress reduction, stimulating social cohesion, providing opportunities for physical activity, and buffering the effects of air pollution (106). Spending at least 120 min in contact with nature during a regular week was associated with a greater likelihood of reporting better SRH and Subjective well-being in a sample representative of the English population, with even greater benefits for longer exposure times (107). Studies have also demonstrated that the health-related benefits of nature contact can apply to people from various cultures and ethnicities (108), with natural environments serving as a protective factor for the health and well-being of immigrant populations (109). The negative association of SRH with nature experiences in our study may be explained by the concomitance of other factors and needs further investigation.

## Strengths and Limitations

To the best of the authors' knowledge, this is one of the few studies investigating health and, specifically, SRH in the context of intra-EEA immigration. The findings of this study provide new knowledge about Italian immigrants in contemporary time, a largely under-researched group. The study reflects the conditions of the Italians living in Norway and cannot be generalized to Italian migration to other countries as the socio-demographic and migration-related characteristics of Italians migrating to Norway may be different from Italians migrating elsewhere (97). The Italians living in Norway have a particularly high level of education. This may reflect a new trend in Italian immigration, but also the appeal of Norway as a destination country for voluntary migrants.

The study has several limitations. One of the limitations of the study is that we could not have direct access to a complete and updated contact list of Italian residents in Norway, which hindered us from performing a randomized or stratified sampling. However, the resampled dataset used in this study, which balanced the original dataset with respect to the profile of key socio-demographic characteristics of the target population (64), allowed us to enhance the representativeness of our original sample. In this paper, it was used the ADASYN approach that creates artificial examples reflecting the pattern of the actual respondents' answers resulting in not being distinguishable from them. The main limitation of such oversampling approach is that it could highlight patterns in the data that could overestimate the analytics' results. Fortunately, the dataset used in this study does not appear to have suffered from this problem.

By using a machine learning approach, we were able to produce a predictive model of SRH that showed relatively high precision. Such an approach allows to detect complex linear and non-linear patterns within the data, with a complete overview of the relationship between the dependent variable (SRH) and the independent variables. This approach presents, however, some limitations. Firstly the findings could be difficult to be interpreted by readers. In the attempt to address this issue, we provided a thorough explanation of the analytical process throughout the text. Another limitation of this approach may reside in its largely inductive nature, which may result in outcomes difficult to explain or apprise in light of existing health theories. On the other hand, this inductive nature can help detect relevant variables not commonly used in studies with deductive analytical approaches. Finally, due to the relatively small sample size, the machine learning models, which are usually adopted in the context of big-data analysis, may have failed in detecting all the possible patterns in the dataset.

By focusing on SRH as an indicator of health, an instrument commonly used in national and international surveys (such as the Eurostat), we could compare the ratings of SRH among Italian immigrants in Norway with the Norwegian and the Italian general populations. SRH is largely used in population studies, showing high reliability (11) and validly concerning objective indicators such as mortality (13, 110). This instrument was appropriate for our study as our aim was not to detect specific morbidity trends, but rather a more comprehensive measure of the Italian immigrants' health. However, SHR has been criticized for being culturally sensitive and therefore comparisons among groups of individuals with different cultural backgrounds need to be taken with caution (25, 40).

## Conclusions

Mobility across Europe is increasingly becoming part of the life of the European population. Most studies on immigrants' health have focused on groups coming from non-EEA countries. Therefore, knowledge of migration health in the context of intra-EEA is needed. The Italians living in Norway can to a large extent be considered as part of a “new mobility” characterized by high educated people, navigating in a globalized world of work and study opportunities. Our study shows that Italian immigrants in Norway tend to have similar SHR compared with the Norwegian general population, with the majority of the immigrants perceiving that the migration process did not have a relevant impact on their health. This finding is in contrast with previous studies indicating deterioration of health after migration and suggests the need of gathering evidence of the impact of migration on health among diverse populations groups. Still, given the high education of the Italians in our study, we could have expected higher SRH levels compared to both the Norwegian and the Italian general populations. The fact that SRH of the Italian immigrants is the same as the overall population both in Italy and in Norway can indicate that Italians living in Norway may have lost the advantage given by a high educational level in the migration process. This is corroborated by the consistent group of Italian immigrants perceiving that their health would have been better had they continued living in Italy. Altogether, this suggests the presence of health challenges also in this group of highly educated immigrants. Trust in people and in the health sector, the acculturation process, alongside with socio-demographic characteristics and health-related behaviors, are important in shaping the health of this population group. Although this study relates specifically to Italian immigrants, the findings may be extended to other immigrant populations in similar contexts. More research on SRH in the context of intra-EEA immigration is recommended.

## Data Availability Statement

The raw data supporting the conclusions of this article will be made available by the authors, without undue reservation.

## Ethics Statement

The studies involving human participants were reviewed and approved by University of Fortaleza Ethics Committee. The patients/participants provided their written informed consent to participate in this study.

## Author Contributions

LT was the overall project coordinator for the Mens Sana in Corpore Sano project, contributed substantially to the design of the study, drafted the introduction, discussion, conclusion, and led the team of authors. AR performed the resampling, statistical analysis, and drafter the relative parts in the methods. MM provide substantial intellectual contribution in the wiring of the introduction section, especially with respect to the historical perspective, and drafted the section on the Italians migration in Norway. GC was together with LT initiator of the project Mens Sana in Corpore Sano, and contributed substantially to the design of the study, statistical analysis, drafting of the results section, and revision of the manuscript. All authors contributed to the article and approved the submitted version.

## Funding

This study was part of a larger project Mens Sana in Corpore Sano, as part of the activities of the project Scienze senza confini (Science without borders) and financed by the Italian Ministry of Foreign Affairs through COMITES Oslo. In particular, this study received some minor funding for administrative expenses and to arrange dissemination seminars. None of the authors is employed or an elected member of COMITES Oslo or the Italian Ministry of Foreign Affairs, nor has received direct funding to conduct this research. The authors' participation in the research activity was entirely funded by their respective institutions. This work is supported by the European Community's H2020 Program under the funding scheme H2020-INFRAIA-2019-1 Research Infrastructures grant agreement 871042, www.sobigdata.eu, accessed on 2 November 2021, SoBigData++: European Integrated Infrastructure for Social Mining and Big Data Analytics. The funders had no role in study design, data collection and analysis, decision to publish, or preparation of the manuscript.

## Conflict of Interest

The authors declare that the research was conducted in the absence of any commercial or financial relationships that could be construed as a potential conflict of interest.

## Publisher's Note

All claims expressed in this article are solely those of the authors and do not necessarily represent those of their affiliated organizations, or those of the publisher, the editors and the reviewers. Any product that may be evaluated in this article, or claim that may be made by its manufacturer, is not guaranteed or endorsed by the publisher.
