# Supplementary material for: Self-Rated Health Among Italian Immigrants Living in Norway: A Cross-Sectional Study
Source: Front Public Health. 2022 Jun 1;10:837728. doi: 10.3389/fpubh.2022.837728 (PMC9198252; doi:10.3389/fpubh.2022.837728)
Supplement: Supplementary file 1 [file Data_Sheet_1.PDF]

***Supplementary material*.**

List of independent variables (predictors) included in the machine learning analysis.

Age: Assessed by asking the year of birth, which was then converted in age (years)

Belief about future: This measure indicate respondents' belief regarding their future place of residence, and was assessed by asking the respondent to select the option that most fit them among the following: 1) “I plan to live in Norway for a relatively short period, and the return to Italy”; 2) “For now, my life is in Norway, but I think I will move back to Italy when I'll be old/after I go to pension”; 3) “I think I'll spend the rest of my life in Norway”; 4) “I don't think I'll live in Norway for the rest of my life, but I don't think I'll move back to Italy either; I rather think I'll move in some other country”; 5) “I don't know”. To facilitate its inclusion in the analysis, the variable was dichotomized as 0 = “Plan to move again/I don't know” and 1 = “Spend the rest of one's life in Norway”.

Contact with good friends: Assessed through the item “How often do you spend time with people that you value as good friends?”. The respondent was asked to select the response options that best fit them among the following: 1) "Never/less than once a year", 2) "About once a year", 3) "Some times a year", 4) "About once a month", 5) "About once a week", 6) "Almost every day"

Contacts with Italian relatives: Assessed through the item “How often do you have contacts with your Italian family of origin?”. The respondent was asked to select the response options that best fit them among the following: 1) "Never/less than once a year", 2) "About once a year", 3) "Some times a year", 4) "About once a month", 5) "About once a week", 6) "Almost every day"

Educational levels: 1) Primary or lower, 2) High school, 3) B.A. degree, 4) M.A. degree or equivalent, 5) Doctoral degree (highest completed)

Empowerment relative to communication: This was the fourth item previously used in Finbråten et al. (2020), which was used individually as it showed poor consistency with the other three: “Actively participate in the dialogue with health personnel, asking questions and expressing the willingness of monitor my own health.”

Empowerment relative to health behaviours: Empowerment is defined as “a process through which people gain greater control over decisions and actions affecting their health” (Nutbeam & Kickbusch, 1998). As an indicator of empowerment, three of the four items previously used in Finbråten et al. (2020) were employed, which asked to rate, on a 4-points scale (1 = “very difficult”; 2 = “Difficult”; 3 = “Easy”; 4 = “Very easy”), the following statements: 1) “take control of your own health in daily life”, 2) “implement goals related to your own health”, and 3) “take responsibility for your own physical and mental health by being physical active, eating healthily and being social.” The Internal consistency of the instrument was adequate ($\alpha = 0.75$).

Food habits: The extent to which the respondents perceived that, all in all, their food habits were healthy was assessed by asking the participants to rate their agreement (1 = “Not at all”; 5 = “Very agree”) with the statement “My food habits are healthy”.

Gender: Male or female

Health literacy: Health literacy “entails people’s knowledge, motivation and competences to access, understand, appraise and apply health information in order to make judgements and take decisions in everyday life concerning health care, disease prevention and health promotion to maintain or improve quality of life during the life course” (Kickbusch et al., 2013). Assessed using the Short Version of the European Health Literacy Survey Questionnaire (HLS-Q12; Finbråten et al., 2018), which consists of 12 items asking to rate, on a 4-points scale (1 = “very difficult”; 2 = “Difficult”; 3 = “Easy”; 4 = “Very easy”), different behaviours such as “find information on treatments of illnesses that concern you” and “judge if the information in the media on health risks is reliable (TV, internet or other media)”.). The resulting total score ranges between 12 and 48, with ratings below 26 indicating ‘inadequate’ health literacy, ratings between 27 and 32 indicating ‘marginal’ health literacy, ratings between 33 and 38 indicating ‘intermediate’ health literacy, and ratings above 39 indicating ‘advanced’ health literacy. The internal consistency of the instrument was adequate ($\alpha = 0.83$

Identifying as a Norwegian: Assessed through the item "If you should define your identity in terms of nationality, would you say that (select the most fitting response option)…" with the response options being: 1) "I always identify as an Italian", 2) "I predominantly identify as an Italian", 3) "I identify as both, an Italian and a Norwegian, in equal extents", 4) "I predominantly identify as a Norwegian", 5) "I always identify as a Norwegian". An additional option "I don't know" was also provided, but excluded from further analyses.

Identifying as an immigrant: Assessed through the item: "Do you think that the term 'immigrant' fits your situation as Italian in Norway?". Response options: 1) "No, absolutely not", 2) "Only in part", 3) "Yes, absolutely yes". An additional option "I don't know" was also provided, but excluded from further analyses.

Italian friends: Assessed through the item "How many of your friends, here in Norway, are of Italian origin?", with the response options being: 1) "None", 2) "Less than half", 3) "About half", 4) "More than half", 5) "All of them"

Language proficiency: Assessed through the item “How would you rate your Norwegian-language skills?”, which was rated through a Likert scale: 1) "Very poor", 2) "Poor", 3) "Intermediate", 4) "Good", 5) "Very good"

Living arrangement: This was a multiple-choice item asking the respondents to report with whom they lived. Several options were provided –the respondents could select more than one option. The options were subsequently merged in the following categories (each of these categories was used as an independent, dichotomous, predictor):

- Living alone
- Living with children (own or of the partner's)
- Living with partner

Motive for moving to Norway: This was a multiple-choice item asking the respondents to select from a list the reasons/motives that they felt were important for taking the decision of moving to Norway -the respondents could select more than one option. The options were subsequently merged in the following categories (each of these categories was used as an independent, dichotomous, predictor):

- Family-related reasons (i.e., following a partner, family member/s, or re-uniting with partner or other family member/s)

- Job-related reasons (i.e., having received a job offer or looking for job opportunities)
- Other reasons (not mentioned in previous options)

Nature restoration: Given its well-known salutogenic effects (van den Bosch & Bird, 2018), as well as its cultural value in Norway (Gelter, 2000), as an indicator of the respondents' opportunities to engage in restorative nature, they were asked to rate their agreement (1 = "Not at all"; 4 = "Absolutely agree") with the statement "I often find myself immersed in nature quietness".

Occupational situation: Whether the respondent was 1) "unemployed, 2) "a student", 3) "engaged in occasional occupation", 4) "self-employed", 5) "hired by the piece", 6) "employed with a term contract", 7) "employed with a permanent contract", 8) "other. " The respondent was asked to select which of these options best described their job situation.

People with whom one moved to Norway: This was a multiple-choice item asking the respondents to report with whom they moved to Norway. Several options were provided -the respondents could select more than one option. The options were subsequently merged in the following categories (each of these categories was used as an independent, dichotomous, predictor):

- *Moved alone*
- *Moved with family* (partner or other family member/s, whether they travelled together or if the respondent moved to reunite with the partner of family member/s)
- *Moved with others* (not mentioned in previous options)

Perceived impact on different aspects of health – These variable provided an indication of the extent to which, all in all, the respondents perceived that different aspect of their health and health-related behaviour were influenced (positively or negatively) by the migration process. This was measured with a single item inquiring the following: "Imagine that you did not move to Norway and, instead, continued to live in Italy. What of the following statements would better reflect your [specific health aspect] in such a hypothetical circumstance?" The response options were: "My [specific health aspect] would have been better in Italy than now in Norway," "My [specific health aspect] would have been more or less the same in Italy as I it is now in Norway," and "My [specific health aspect] would have been worse in Italy than pit is now in Norway." An additional response option "I don't know" was also included, but was excluded from further analyses. The following specific health aspects were included:

- *... on food habits*
- *... on physical activity habits*
- *... on social relationship*

Region of residence: Whether or not the respondent lived in Oslo/Akershus (i.e., the most urbanized and densely populated region of Norway)

Satisfaction with occupation: The extent to which the respondent perceived that his or her occupation was adequate with respect to his or her educational background, which was assessed by asking the respondent to select the response option that best described their levels of satisfaction among the following: 1) "My current occupation is unsatisfactory considering my educational background", 2) I have a satisfactory occupation, although it does not match my educational background, 3) My occupation is satisfactory and matches well my educational background; 4) Considering my educational background, I am highly satisfied

with my occupation. An additional response option "at the moment I am unemployed" was also provided, but excluded from further analyses.

Tobacco usage: This was assessed through the following item: “Do you smoke or use tobacco (e.g., snus)?”, with the response options being: 1) “Yes, daily”; 2) Yes, occasionally”; 3) “No”.

Trust in people: This was assessed through the following Item: “On a scale from 0 to 10, how would say that one can trust most people, or that one should always better be careful in trusting others?”. The response was provided on a visual scale ranging where 0 was the lowest level of trust and 10 the highest.

Trust in the Norwegian Health System: This was assessed through the following Item: “On a scale from 0 to 10, how would you rate your level of trust in the Norwegian health system, including doctor and other health personnel?”. The response was provided on a visual scale ranging where 0 was the lowest level of trust and 10 the highest.

Weekly physical activity: This variable provided an indication of the extent to which the respondents engaged in insufficient or sufficient moderate-to-vigorous physical activity levels, broadly in line with the World Health Organization’s recommendations (WHO, 2020). First, the following definition was presented: “Think about your physical activity habits in the course of the past 12 months. By the term “physical activity” we intend any bodily movement that, for at least 10-consecutive minutes, makes your heart beat faster and your breathing harder. This can include, for example, structured exercise but also activities during working- or school hours, house chores, going for a stroll, or if you walk or bike to/from work.” Subsequently, the following question was presented “During a regular week, all in all, how much time do you spend doing physical activity?” with the response options being “I never engage in any physical activity during a regular week,” “Less than 2.5 h,” “Between 2.5 and 5 h,” and “More than 5 h.”

Years of permanence in Norway: Assessed by asking to report the year in which the respondents settled permanently in Norway, which was then converted in the number of years since moving.

References

Barstad, A. (2018). Livskvalitet blant innvandrere. En analyse basert på Levekårsundersøkelsen blant personer med innvandrerbakgrunn 2016 [Quality of life among immigrants. An analysis of the Survey on living conditions among persons with an immigrant background 2016] (Report No. 2018/31). Statistics Norway. https://www.ssb.no/en/sosiale-forhold-og-kriminalitet/artikler-og-publikasjoner/quality-of-life-among-immigrants.an-analysis-of-the-survey-on-living-conditions-among-persons-with-an-immigrant-background-2016.

Finbråten, H. S., Guttersrud, Ø., Nordström, G., Pettersen, K. S., Trollvik, A., and Wilde-Larsson, B. (2020). Explaining variance in health literacy among people with type 2 diabetes: the association between health literacy and health behaviour and empowerment. BMC Public Health, 20(1), 161.

Finbråten, H. S., Wilde-Larsson, B., Nordström, G., Pettersen, K. S., Trollvik, A., and Guttersrud, Ø. (2018). Establishing the HLS-Q12 short version of the European Health

Literacy Survey Questionnaire: latent trait analyses applying Rasch modelling and confirmatory factor analysis. BMC Health Services Research, 18(1), 1-17.

Gelter, H. (2000). Friluftsliv: The Scandinavian philosophy of outdoor life. Canadian Journal of Environmental Education (CJEE), 5(1), 77-92.

Kickbusch, I., Pelikan, J. M., Apfel, F., and Tsouros, A. (2013). Health literacy. WHO Regional Office for Europe.

Nutbeam, D., & Kickbusch, I. (1998). Health promotion glossary. Health promotion international, 13(4), 349-364.

Van den Bosch, M., & Bird, W. (Eds.). (2018). Oxford textbook of nature and public health: The role of nature in improving the health of a population. Oxford University Press.

World Health Organization. WHO Guidelines on Physical Activity Sedentary Behaviour. Geneva: World Health Organization (2020).

Oversampling

A comparison of basic sociodemographic characteristics (gender, age, educational level, and region of residence) of the sample with figures provided by national registers, such as AIRE (1), revealed that our sample was not fully representative of the overall population of Italian immigrants in Norway. The sample had a larger proportion of women, mid-aged individuals, people with a higher educational level, and people living in the region of Oslo-Akershus, which is the most densely populated and urbanized area of Norway (Table 1). In order to enhance the sample's representativeness, the dataset was oversampled in accordance with the proportion of key sociodemographic characteristics of the reference population, which were provided by the Italian Embassy as based on the AIRE registry. To this aim, Adaptive Synthetic Sampling Method for Imbalanced Data (ADASYN) was applied by using the publicly available Python package imblearn (http://scikit-learn.org/imbalanced-learn). ADASYN automatically estimates the needed number of synthetic cases according to a density distribution defined by the expected proportions of given variable in the reference population of Italians in Norway. In particular, considering a vector case x_i, a new vector case x_{new} will be generated considering its k 3 nearest-neighbors (x_{zi}) as follow:

$$Xnew=xi+\lambda * (xzi-xi)Xnew=xi+\lambda * (xzi\text{-}xi)$$

where λ is a random number between [0,1]. For this study, the oversampling process was performed on 2-factors, i.e., age and educational level. The resampled dataset reflects an acceptable distribution of key sociodemographic variables with relatively limited inclusion of synthetic cases (additional synthetic cases = 231; overall n = 531; Table 1).

Table 1.
Data distribution of the reference population, the original dataset, and the resampled dataset.

**Sociodemographic characteristics**	**Italians in Norway (N = 3474)[a]**	**Mens Sana in Corpore Sano dataset (n = 321)**	**Resampled dataset (n = 531)**
**Gender**			
Male	63%	47%	61%
Female	37%	53%	39%
**Age**			
18-30 y	23%	14%	19%
31-50 y	54%	71%	56%
>50 y	23%	15%	25%
**Educational level (highest completed)**			
Up to secondary upper-level school	40%	18%	39%
Bachelor or higher	60%	82%	61%
**Region of residence**			
North	3%	7%	6%
Center	7%	11%	12%
West	20%	14%	19%
Oslo/Akershus	53%	60%	57%
Other eastern regions	13%	8%	6%
South	3%	1%	1%

[a] Based on the AIRE register; information provided by the Italian Embassy in Norway (1).
[b] Resampling was based on Age and Educational level
Region of residence: North = Finnmark, Troms, Nordland; Center = N. & S.Trøndelag, Møre og Romsdal; West = Sogn og Fjordane, Hordaland, Rogaland; Other eastern regions = Telemark, Buskerud, Vestfold, Østfold, Oppland, Hedmark; Sout = V. Agder, A. Agder.

References:

1) Ambasciata d'Italia Oslo. Italiani in Norvegia - statistiche aggiornate a luglio 2019 [Italians in Norway – updated statistics July 2019]. Available online at: https://amboslo.esteri.it/ambasciata_oslo/en/ambasciata/news/dall_ambasciata/2018/05/italiani-in-norvegia-statistiche.html (accessed March 31, 2021).

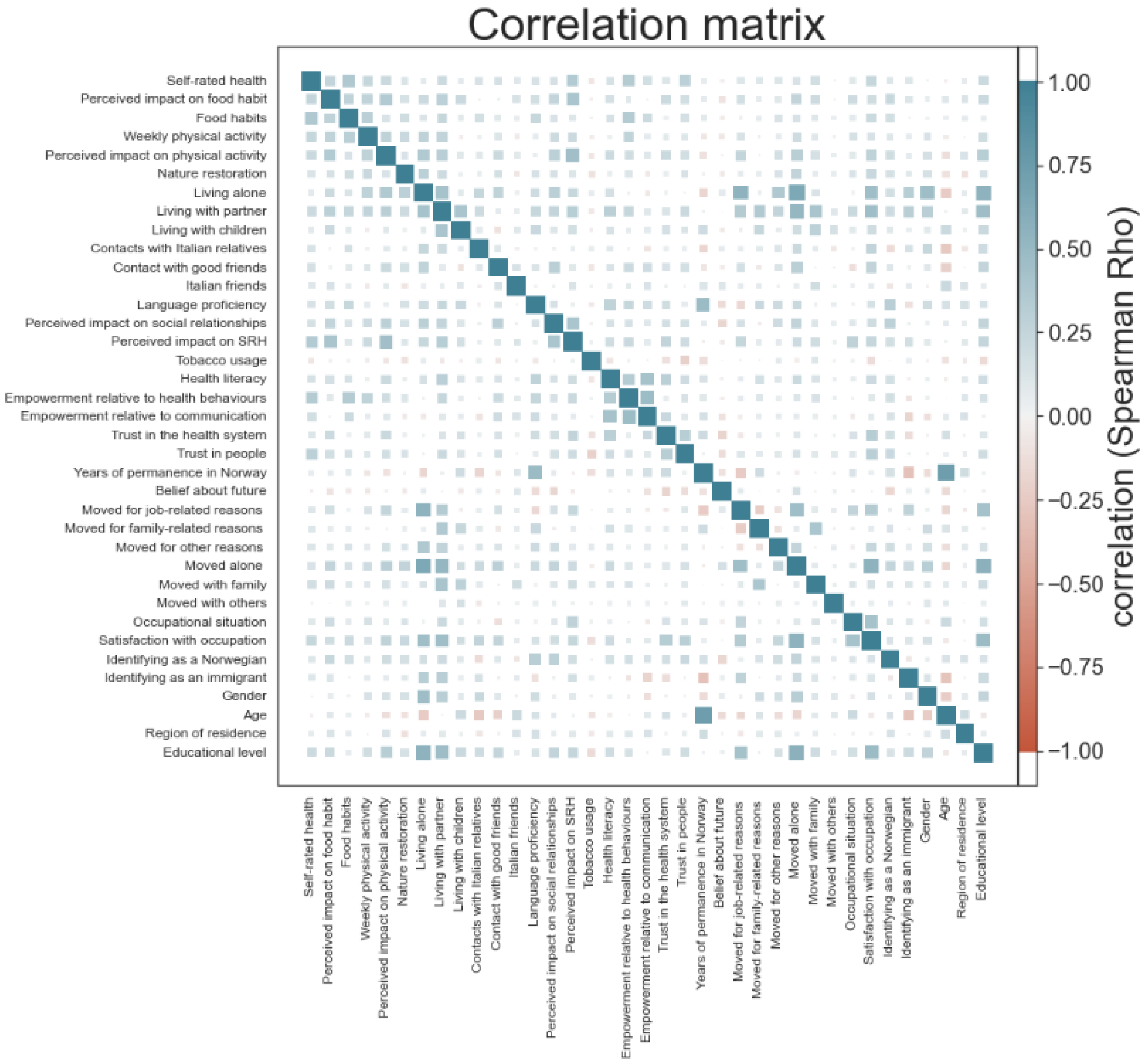

Supplementary Figure 1. Correlation matrix (Spearman's Rank Correlation Coefficient; *rho*) showing significant relationship among variables in the dataset. The dimension of the squares refers to the strength of the relationship between two variables. Moreover, blue and red colours provide the direction of the correlation (i.e., positive and negative, respectively) and, in accordance with the dimension, the darker is the colour the strength is the correlation. Only significant associations (i.e., $df_{(n-2)} = 529$, p-value = 0.05, *rho* > 0.088) are presented.

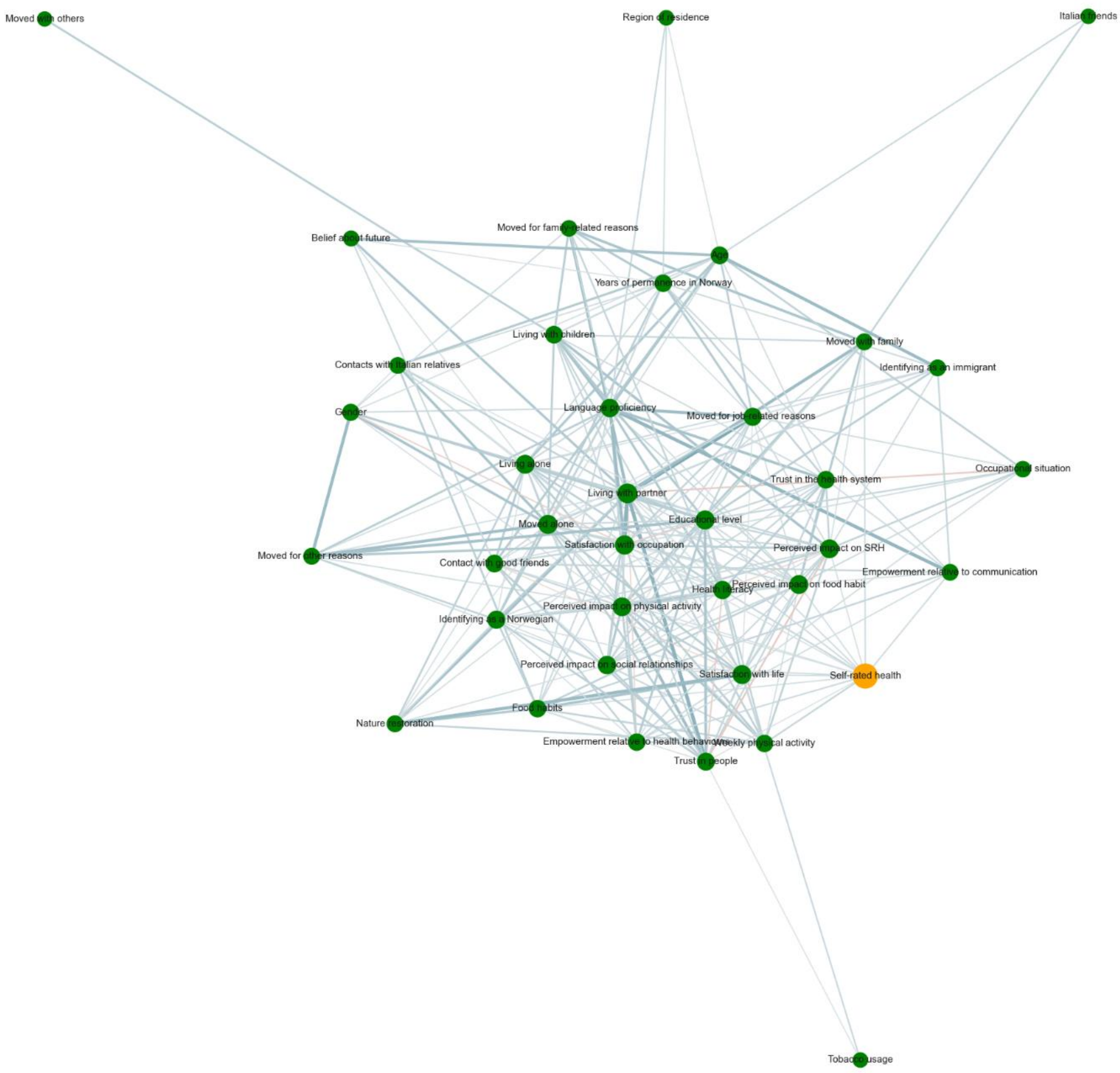

Supplementary Figure 2. Correlation network showing the correlation network space among all the variables used in the study (number of nodes = 38; number of edges = 267; network density = 0.37). The nodes and edges refer to the single variable and the correlation link between two nodes, respectively. The distance between nodes refers to the strength of their correlation. The closer the nodes are, the higher the correlation between the two nodes are. GE feature is highlighted in orange. The colours of the links refers to the correlations between nodes as showed in Supplementary Figure 1.
